# Origin and Population Dynamics of a Novel HIV-1 Subtype G Clade Circulating in Cape Verde and Portugal

**DOI:** 10.1371/journal.pone.0127384

**Published:** 2015-05-20

**Authors:** Isabel Inês M. de Pina-Araujo, Edson Delatorre, Monick L. Guimarães, Mariza G. Morgado, Gonzalo Bello

**Affiliations:** 1 Departamento de Ciência e Tecnologia, Universidade de Cabo Verde, Praia, Santiago, Cabo Verde; 2 Laboratório de AIDS & Imunologia Molecular, Instituto Oswaldo Cruz, FIOCRUZ, Rio de Janeiro, Brazil; National Institute of Health, ITALY

## Abstract

The human immunodeficiency virus type 1 (HIV-1) subtype G is the most prevalent and second most prevalent HIV-1 clade in Cape Verde and Portugal, respectively; but there is no information about the origin and spatiotemporal dispersal pattern of this HIV-1 clade circulating in those countries. To this end, we used Maximum Likelihood and Bayesian coalescent-based methods to analyze a collection of 578 HIV-1 subtype G *pol* sequences sampled throughout Portugal, Cape Verde and 11 other countries from West and Central Africa over a period of 22 years (1992 to 2013). Our analyses indicate that most subtype G sequences from Cape Verde (80%) and Portugal (95%) branched together in a distinct monophyletic cluster (here called G_CV-PT_). The G_CV-PT_ clade probably emerged after a single migration of the virus out of Central Africa into Cape Verde between the late 1970s and the middle 1980s, followed by a rapid dissemination to Portugal a couple of years later. Reconstruction of the demographic history of the G_CV-PT_ clade circulating in Cape Verde and Portugal indicates that this viral clade displayed an initial phase of exponential growth during the 1980s and 1990s, followed by a decline in growth rate since the early 2000s. Our data also indicate that during the exponential growth phase the G_CV-PT_ clade recombined with a preexisting subtype B viral strain circulating in Portugal, originating the CRF14_BG clade that was later disseminated to Spain and Cape Verde. Historical and recent human population movements between Angola, Cape Verde and Portugal probably played a key role in the origin and dispersal of the G_CV-PT_ and CRF14_BG clades.

## Introduction

The global dissemination of human immunodeficiency virus type 1 (HIV-1) group M, the pandemic clade of HIV, resulted from the random exportation out of Central Africa of a few viral strains designated as subtypes (A–D, F–H, J and K) and inter-subtype circulating recombinant forms (CRFs) [1].

Subtype G is the sixth most prevalent HIV-1 clade in the world accounting for nearly 5% of all global infections [2]. This subtype reaches the highest prevalence in some African countries, comprising 30–50% of HIV-1 infections in Cape Verde [3,4] and Nigeria [5–12], and 5–15% of HIV-1 infections in Angola [13–15], Benin [16], Niger [17,18] and Togo [19,20]. A recent study conducted by our group suggests that subtype G most probably emerged in Central Africa around the late 1960s and was rapidly disseminated into the West and West Central African regions [21]. This study showed that basal subtype G lineages (G_CA_) were mostly restricted to Central and West Central African countries. Two subtype G strains, however, gained access to West Africa between the middle and the late 1970s and fueled secondary local outbreaks, leading to the origin of two major subtype G West African clades (G_WA-I_ and G_WA-II_).

Some subtype G strains where also disseminated out of the African continent and the most remarkable example is Portugal where subtype G is the second most prevalent HIV-1 clade (>10%), after subtype B (> 40%) [22–25]. The high prevalence of subtypes B and G in Portugal has also promoted the appearance of different types of B/G recombinant strains, including one circulating recombinant form (CRF14_BG) that was initially identified in Galicia, Northern Spain [26]. According to a previous study, the CRF14_BG probably emerged in Portugal in the early 1990s and later spread to Galicia in the late 1990s as a consequence of the mobility of HIV-infected injecting drug users (IDUs) [27].

Notably, about two-thirds of the subtype G viruses previously described in Portugal were found in individuals from Angola and Cape Verde [23]. These countries are two former Portuguese African colonies that have strong historic links and maintain ongoing relationships with Portugal and displayed a relatively high prevalence of subtype G [3,4,13–15]. The high numbers of immigrants from Angola and Cape Verde who enter Portugal and also those Portuguese returning after living in the former Portuguese African colonies from the 1970s onwards [28], supposes a potential risk for introduction of HIV-1 subtype G strains in Portugal. However, the precise evolutionary relationship between subtype G viruses circulating in Angola, Cape Verde and Portugal remains unknown.

The objective of this study was to reconstruct the phylogenetic relationship, onset date and dissemination routes of the HIV-1 subtype G clades circulating in Angola, Cape Verde and Portugal. To this end, we used Maximum Likelihood and Bayesian coalescent-based approaches to analyze 578 HIV-1 subtype G *pol* sequences isolated from Portugal, West Africa and Central Africa over a period of 22 years (1992 to 2013).

## Materials and Methods

### Sequence dataset

All HIV-1 subtype G *pol* sequences from Portugal and West/Central African countries that covered the entire protease and partial reverse transcriptase (PR/RT) regions (nt 2253–3272 relative to HXB2 clone) and for which the sampling year was known, were downloaded from the Los Alamos HIV Sequence Database (www.hiv.lanl.gov) by December 2014. The same procedure was adopted to obtain the subtype G *pol* fragment of all CRF14_BG sequences from Portugal and Spain (the main countries were this CRF circulates) with full-length genome characterization up to date. The subtype assignment of all sequences was confirmed by REGA HIV subtyping tool v.2 [29] and bootscanning analysis. In bootscanning analyses, supporting branching of query sequences with HIV-1 group M subtype reference sequences was determined in Neighbor-Joining trees constructed with the Kimura two-parameter model, within a 250bp window moving in steps of 10 bases, using Simplot software v.3.5.1 [30]. Sequences with incorrect classification, multiple sequences from the same individual and sequences from countries poorly represented (*n* < 4 sequences) were removed, resulting in a final data set of 578 HIV-1 subtype G *pol* sequences (Table 1). Sequence’s GenBank accession numbers are available in S1 Table. All codon positions known to be associated with major antiretroviral drug resistance were maintained in the final alignment because phylogenetic trees constructed on alignments with or without such positions resulted in the same overall topology (data not shown). The presence of phylogenetic signal and substitution saturation in our datasets was investigated by: 1) using the likelihood mapping analysis [31] performed with TREE-PUZZLE v5.2 program [32] implemented in the online web platform Mobyle@Pasteur v1.5 [33], and 2) plotting the observed number of transitions and transversions against genetic distance for each pairwise comparison, calculated under the GTR+I+G nucleotide substitution model using DAMBE v5.3 program [34].

**Table 1:** HIV-1 subtype G *pol* dataset.

Los Alamos classification	Region	Country	*N*	Sampling interval
Subtype G	West Africa	Benin	15	2004–2009
		Ghana	9	2002–2010
		Cape Verde	60	2005–2011
		Nigeria	223	1992–2013
		Senegal	12	1998–2010
		Togo	28	2006–2011
	West Central Africa	Cameroon	62	1997–2012
		Gabon	6	2000–2008
		Equatorial Guinea	4	2005–2009
	Central Africa	Angola	20	1997–2010
		DRC	12	1993–2007
		Republic of Congo	8	2003
	Europe	Portugal	107	1998–2008
CRF14_BG	Europe	Portugal	2^a^	1998–2008
		Spain	10^a^	1999–2005

^a^ Subtype G *pol* fragments recovered from full-length CRF14_BG reference sequences.

### Identification of major HIV-1 subtype G clades

Major HIV-1 subtype G clades were identified by Maximum Likelihood (ML) phylogenetic analysis. A ML phylogeny was constructed with the PhyML 3.0 program [35] using an online web server [36]. The ML tree was inferred under the GTR+I+G nucleotide substitution model as recommended by the jModeltest program [37], the heuristic tree search was performed using the SPR branch-swapping algorithm and branch support was calculated with the approximate likelihood-ratio (aLRT) SH-like test [38].

### Analysis of spatiotemporal dispersion pattern and demographic history

The evolutionary rate (*μ*, nucleotide substitutions per site per year, subst./site/year), the age of the most recent common ancestor (T_MRCA_, years), the ancestral geographic movements, and the mode and rate (*r*, years-1) of population growth of HIV-1 subtype G clades were jointly estimated using the Bayesian Markov Chain Monte Carlo (MCMC) approach as implemented in BEAST v1.8 [39,40] with BEAGLE to improve run-time [41]. Analyses were performed under a GTR+I+G nucleotide substitution model. The temporal scale of the evolutionary process was estimated from the sampling dates of the sequences using a relaxed uncorrelated lognormal molecular clock model and a uniform prior on clock rate (1.5–3.0 x 10^–3^ subst/site/year) [42]. Migration events throughout the phylogenetic history were inferred using a reversible discrete Bayesian phylogeographic model [43], in which all possible reversible exchange rates between locations were equally likely, and a CTMC rate reference prior [44]. Changes in effective population size through time were initially estimated using a flexible Bayesian Skyline coalescent model [45] and estimates of the population growth rate were subsequently obtained using the parametric model (logistic, exponential or expansion) that provided the best fit to the demographic signal contained in datasets. Comparison between demographic models was performed using the log marginal likelihood (ML) estimation based on path sampling (PS) and stepping-stone sampling (SS) methods [46]. MCMC chains were run for 10–100 x 10^6^ generations. Adequate chain mixing and uncertainty in parameter estimates were assessed by calculating the Effective Sample Size (ESS) and the 95% Highest Probability Density (HPD) values, respectively, using the TRACER v1.6 program [47]. Maximum clade credibility (MCC) trees were summarized from the posterior distribution of trees with TreeAnnotator and visualized with FigTree v1.4.0 [48].

## Results

### Identification of major HIV-1 subtype G clades

**Fig 1:**
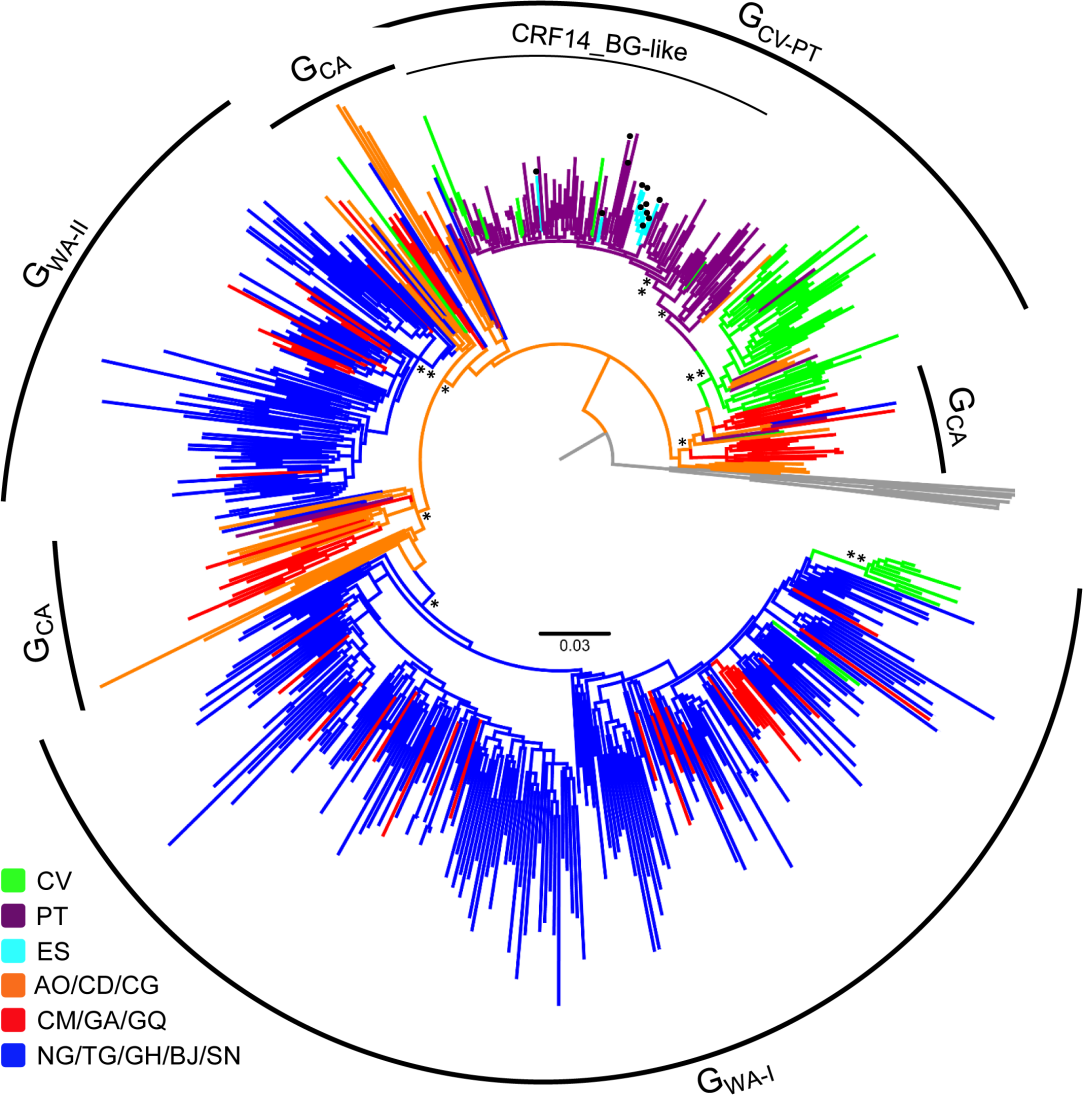
ML tree of the HIV-1 subtype G *pol* PR/RT sequences (~1,000 nt) from Central Africa, West Africa, Portugal and Spain. Branches are colored according to the geographic origin of each sequence as indicated in the legend (bottom left). Arcs indicate the positions of major subtype G clades circulating in Central Africa (G_CA_), West Africa (G_WA-I_ and G_WA-II_) and Cape Verde/Portugal (G_CV-PT_) and the position of the subclade that comprises all CRF14_BG reference sequences (CRF14_BG-like). Black dots indicate the positions of the reference sequences classified as CRF14_BG based on full-length genome analysis. Asterisks point to key nodes with relatively high (*, *a*LRT > 0.80) and high (**, *a*LRT > 0.90) support. The tree was rooted using HIV-1 subtype A-D reference sequences. The branch lengths are drawn to scale with the bar at the center indicating nucleotide substitutions per site. AO/CD/CG: Angola/Democratic Republic of Congo/Republic of Congo; CM/GA/GQ: Cameroon/Gabon/Equatorial Guinea; NG/TG/GH/BJ/SN: Nigeria/Togo/Ghana/Benin/ Senegal; CV: Cape Verde; PT: Portugal; ES: Spain.

**Fig 2:**
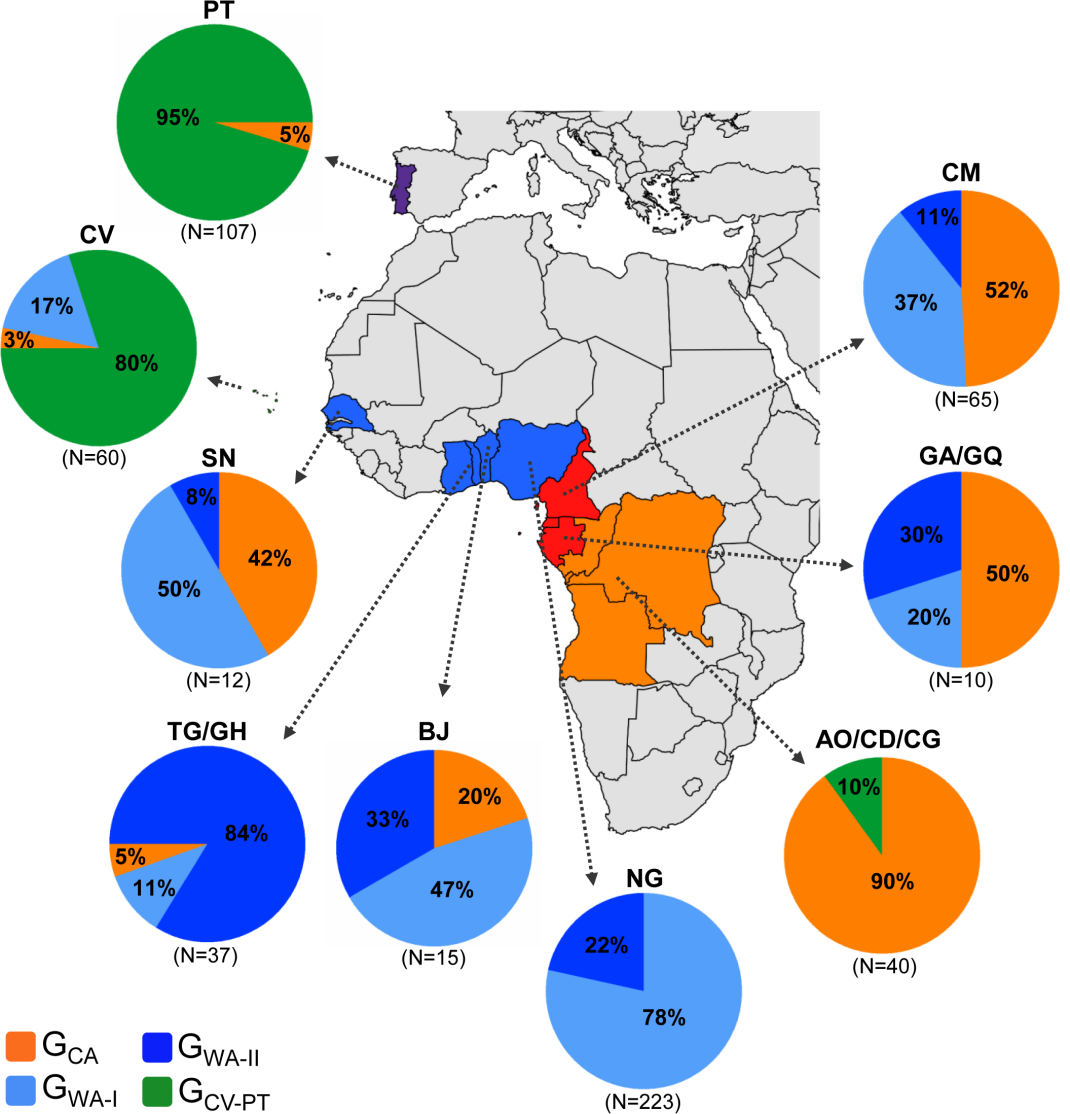
Prevalence of G_CV-PT_, G_WA-I_, G_WA-II_ and G_CA_ clades among HIV-1 subtype G infected individuals from different countries, estimated from phylogenetic analyses presented in Fig 1. The total number of subtype G sequences analyzed in each locality is indicated. Each clade is represented by a color as indicated at the legend. AO/CD/CG: Angola/Democratic Republic of Congo/Republic of Congo; BJ: Benin; CM: Cameroon; CV: Cape Verde; GA/GQ: Gabon/Equatorial Guinea; NG: Nigeria; PT: Portugal; TG/GH: Togo/Ghana; SN: Senegal.

The likelihood mapping analysis and the transitions/transversions versus divergence plots indicates that all datasets used in our study retained enough phylogenetic signal for consistent phylogenetic inferences and no evidence of substitution saturation (S1 Fig). The ML phylogenetic tree of 578 HIV-1 subtype G *pol* sequences (566 classified as subtype G and 12 classified as CRF14_BG in the Los Alamos HIV Sequence Database) isolated in Portugal, Spain and 12 countries from West and Central Africa between 1992 and 2013 (Table 1) points to a clear phylogeographic subdivision of viral strains (Fig 1). Subtype G sequences from continental western African countries branched mostly in two large monophyletic clades (G_WA-I_ and G_WA-II_) that were nested among the most basal clades from Central and West Central Africa (G_CA_), consistent with our previous findings [21]. Although some subtype G sequences from Cape Verde (*n* = 10) also branched within the G_WA-I_ clade; most sequences from this insular West African country (*n* = 48) branched together with most subtype G sequences from Portugal (*n* = 102) in a distinct monophyletic clade (G_CV-PT_) nested among basal G_CA_ lineages. All CRF14_BG sequences and several subtype G *pol* sequences from Portugal (*n* = 78) and Cape Verde (*n* = 7) formed a highly supported sub-cluster (CRF14_BG-like) within the G_CV-PT_ clade. According to the relative prevalence of the distinct subtype G clades, we can describe four basic molecular epidemiologic scenarios (Fig 2 and S2 Table): 1) basal G_CA_ clades are the predominant subtype G lineages circulating in countries from Central (90%) and West-Central (50%) African regions; 2) the G_WA-I_ clade was the predominant lineage detected in Nigeria (78%), Senegal (50%) and Benin (47%); 3) the G_WA-II_ clade predominates in Togo/Ghana (84%); and 4) the G_CV-PT_ clade was the dominant lineage in Cape Verde (80%) and Portugal (95%).

### Spatiotemporal dispersal pattern of the HIV-1 G_CV-PT_ clade

**Fig 3:**
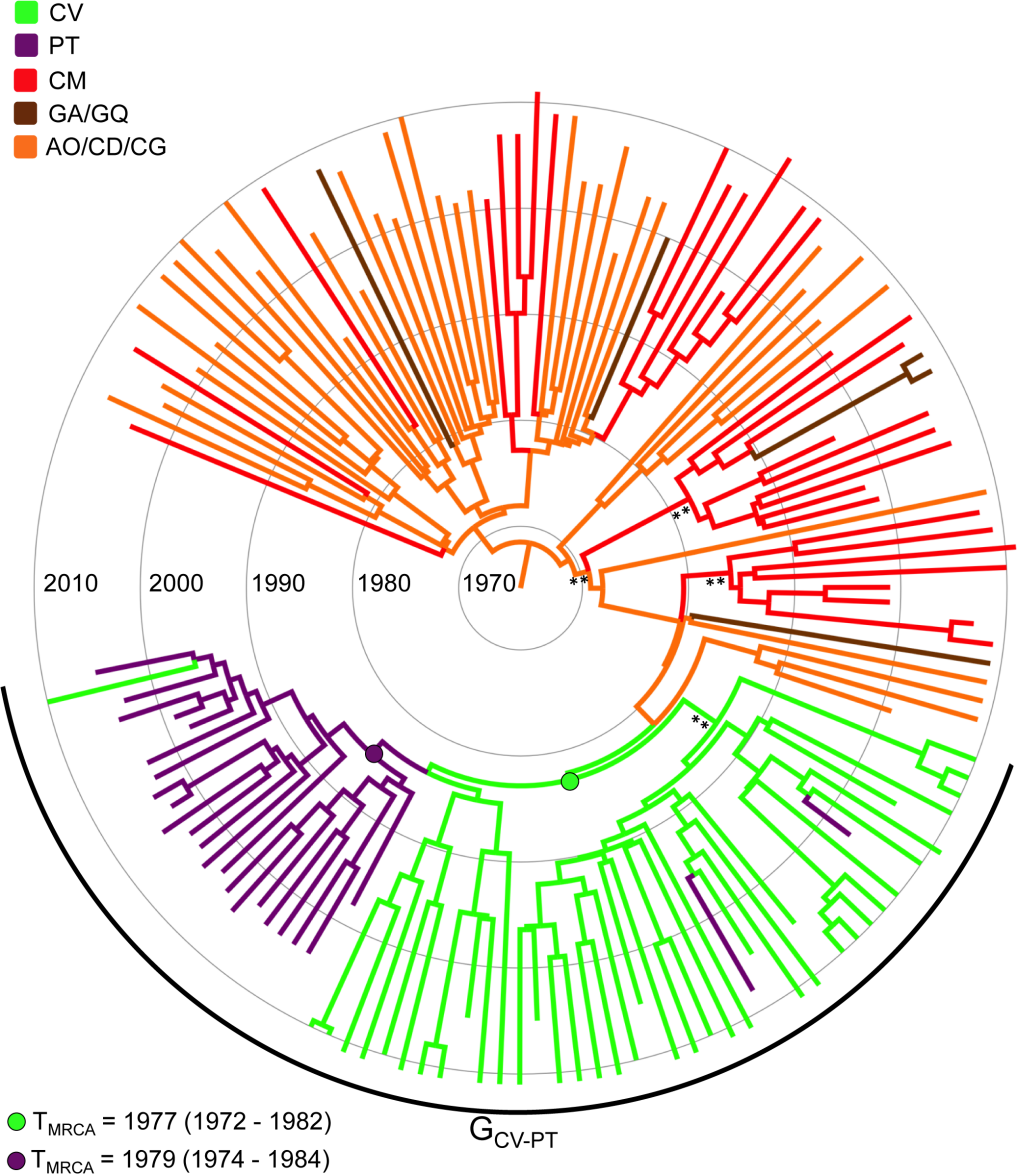
Time-scaled Bayesian MCC tree of HIV-1 subtype G *pol* PR/RT sequences (~1,000 nt) from the G_CA_ and G_CV-PT_ clades. Branches are colored according to the most probable location state of their descendent nodes as indicated in the legend (upper left). The arc indicates the position of the G_CV-PT_ clade. Key nodes corresponding to the MRCA of the Cape Verdean and Portuguese G_CV-PT_ lineages are indicated with circles and the median T_MRCA_ (with the corresponding 95% HPD interval) of each lineage is indicated at the bottom left. Asterisks point to key nodes with relatively high (*, *PP* > 0.80) and high (**, *PP* > 0.90) posterior probability support. Branch lengths are drawn to a scale of years. The tree was automatically rooted under the assumption of a relaxed molecular clock. AO/CD/CG: Angola/ Democratic Republic of Congo /Republic of Congo; CM: Cameroon; CV: Cape Verde; GA/GQ: Gabon/Equatorial Guinea; PT: Portugal.

To reconstruct the subtype G migrations between Africa and Portugal, all subtype G sequences belonging to the G_CV-PT_ clade (excluding the CRF14_BG-like sub-clade) (*n* = 69) were combined with basal G_CA_ strains of Central African origin (*n* = 73). Sequences were divided in six geographical locations, as those neighboring countries from Central and West-Central Africa comprising few samples (*n* < 20) were grouped into the same location state (S3 Table), and subjected to Bayesian phylogeographic analysis According to the Bayesian MCMC analysis, the most probable root location of the subtype G clade was placed in Central Africa (posterior state probability, *PSP* = 1), and the onset date of this clade was estimated to be 1964 (95% HPD: 1937–1978) (Fig 3). Sequences from Cape Verde branched at the base of the G_CV-PT_ clade, whereas most sequences from Portugal branched in a monophyletic cluster nested within the Cape Verdean sequences (Fig 3). This analysis suggests that the G_CV-PT_ clade most probably migrates from Central Africa to Cape Verde (*PSP* = 0.68) at 1977 (95% HPD: 1972–1982) and rapidly moved from Cape Verde to Portugal in 1979 (95% HPD: 1974–1984). A few additional exchanges of the G_CV-PT_ clade between Cape Verde and Portugal were detected at later times (Fig 3).

### Demographic history of the HIV-1 G_CV-PT_ clade

**Fig 4:**
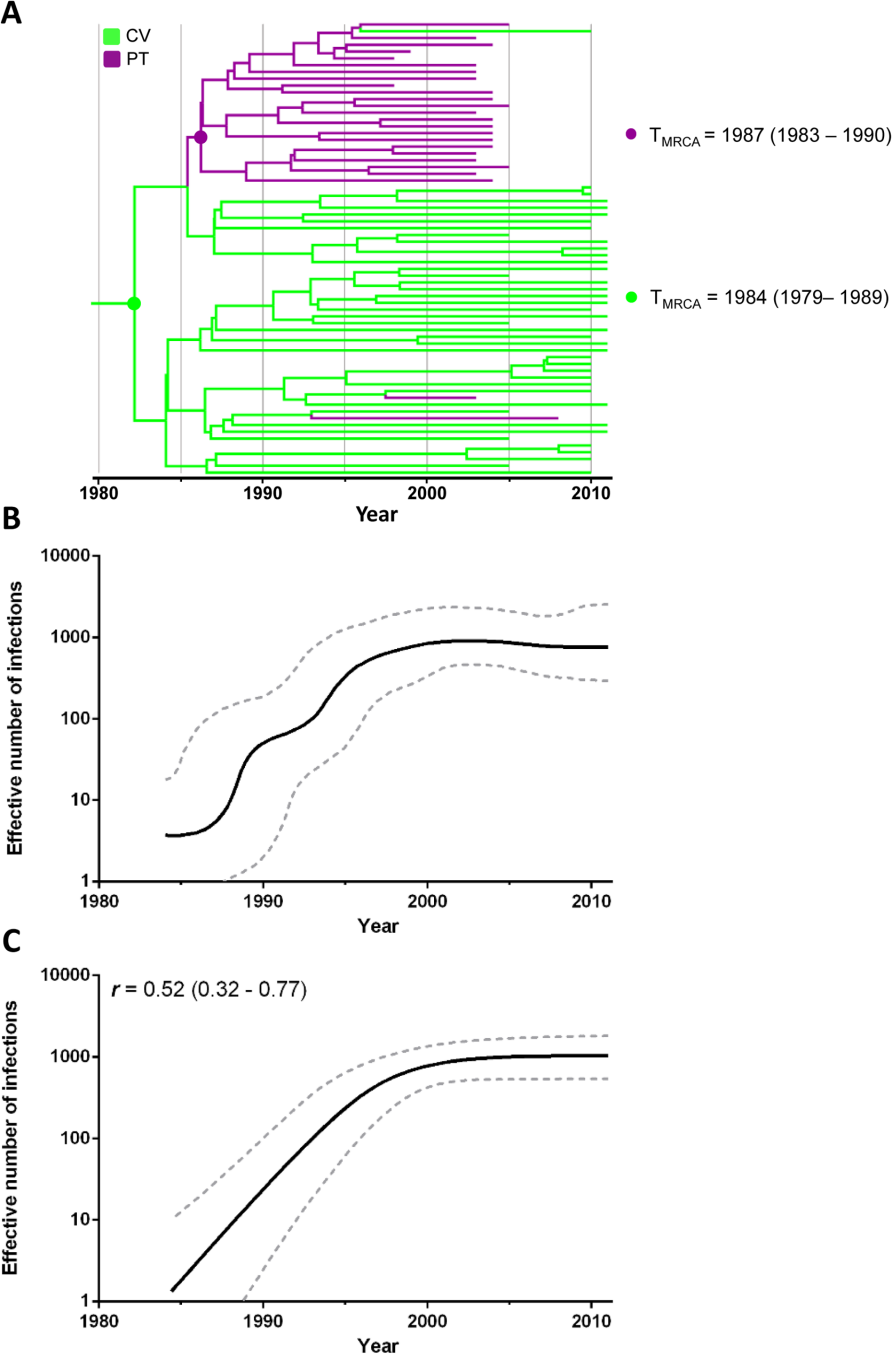
Demographic history of the HIV-1 G_CV-PT_ clade circulating in Cape Verde and Portugal. A) Time-scaled Bayesian MCC tree of the HIV-1 G_CV-PT_ clade. Branches are colored according to the most probable location state of their descendent nodes as indicated in the legend (upper left). Key nodes corresponding to the MRCA of the Cape Verde and Portuguese G_CV-PT_ lineages are indicated with circles and the median T_MRCA_ (with the corresponding 95% HPD interval) of each lineage is indicated at right. Branch lengths are drawn to a scale of years. The tree was automatically rooted under the assumption of a relaxed molecular clock. B and C) Effective number of infections (y-axis; log10 scale) through time (x-axis; calendar years) estimated using Bayesian skyline (B) and logistic growth (C) coalescent models. Median (solid line) and 95% HPD intervals (dashed lines) of the effective number of infections estimated through time are shown in each graphic. The median growth rate (with the corresponding 95% HPD interval) of G_CV-PT_ clade estimated under the logistic growth model is indicated in the upper left corner.

To reconstruct the demographic history of the G_CV-PT_ clade, all subtype G sequences from Cape Verde (*n* = 41) and Portugal (*n* = 24) that branched within this clade (excluding the CRF14_BG-like sub-clade) were selected. In agreement with our previous analysis, most subtype G sequences from Portugal branched in a sub-cluster nested among basal Cape Verdean sequences (Fig 4A). This new analysis, however, supports a relatively more recent time-scale than previous estimations. According to this new analysis, the G_CV-PT_ clade probably arose in Cape Verde (*PSP* = 0.76) in 1984 (95% HPD: 1979–1989) and was rapidly disseminated to Portugal in 1987 (95% HPD: 1983–1990). Two additional migrations of the G_CV-PT_ clade from Cape Verde to Portugal and one migration event from Portugal to Cape Verde were also detected, in agreement with our previous analysis (Fig 4A). The Bayesian skyline plot (BSP) analysis suggests that the G_CV-PT_ clade experienced a fast exponential growth during the 1980s and 1990s, followed by a more recent stabilization since the early 2000s (Fig 4B). According to the logistic growth coalescent model, selected as the best-fit demographic model for the G_CV-PT_ clade (log BF > 10) (S4 Table), the mean growth rate of this subtype G clade was 0.52 year^-1^ (95% HPD: 0.32–0.77 year^-1^) (Fig 4C).

### Origin of the CRF14_BG clade

**Fig 5:**
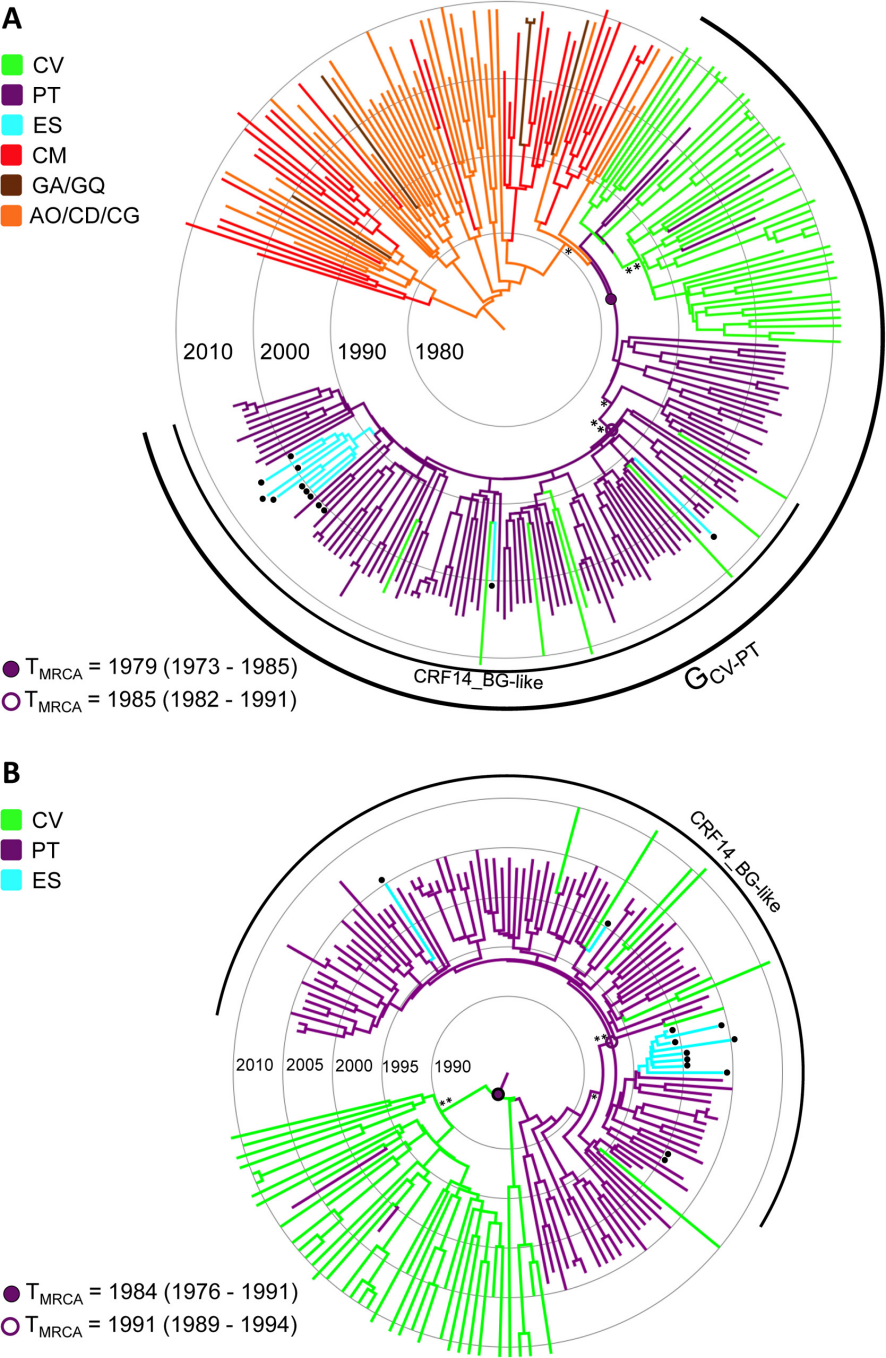
Time-scaled Bayesian MCC tree of HIV-1 subtype G *pol* PR/RT sequences (~1,000 nt) from the G_CA_, G_CV-PT_ and CRF14_BG-like clades. Sequences that branched within the CRF14_BG-like subclade were combined with sequences from G_CA_ and G_CV-PT_ clades (A) or only G_CV-PT_ clade (B). Branches are colored according to the most probable location state of their descendent nodes as indicated in the legend (upper left). Arcs indicate the positions of G_CV-PT_ and CRF14_BG-like clades. Nodes corresponding to the MRCA of those clades are indicated with circles and the median T_MRCA_ (with the corresponding 95% HPD interval) of each clade is indicated at the bottom left. Black dots indicate the position of the CRF14_BG reference sequences. Asterisks point to key nodes with high relatively high (*, *PP* > 0.80) and high (**, *PP* > 0.90) posterior probability support. Branch lengths are drawn to a scale of years. The tree was automatically rooted under the assumption of a relaxed molecular clock. AO/CD/CG: Angola/ Democratic Republic of Congo /Republic of Congo; CM: Cameroon; CV: Cape Verde; GA/GQ: Gabon/Equatorial Guinea; PT: Portugal; ES: Spain.

To investigate the origin of the parental subtype lineage that gave rise to the CRF14_BG, all *pol* sequences that branched within the CRF14_BG-like subclade (*n* = 97) were combined with sequences from clades G_CA_ and G_CV-PT_. The overall topology and temporal structure of the Bayesian MCC trees remains conserved after inclusion of the CRF14_BG-like subclade, but placed most of the posterior root state probability mass of the G_CV-PT_ clade in Portugal (*PSP* = 0.55–0.81) (Fig 5 and S5 Table). Both Bayesian MCMC analyses showed that all CRF14_BG-like sequences formed a well-supported sub-cluster (*PP* > 0.90) nested among basal subtype G Portuguese sequences within the G_CV-PT_ radiation (Fig 5). Those analyses support that the CRF14_BG-like clade most probably arose in Portugal (*PSP* = 1) and was later disseminated at multiple times from Portugal to both Spain and Cape Verde (Fig 5). The *T*_MRCA_ of the CRF14_BG-like clade was traced to 1986 (95% HPD: 1982–1991) when basal G_CA_ strains were included in the analysis (Fig 5A), and to 1991 (95% HPD: 1988–1994) when basal G_CA_ strains were not included (Fig 5B).

## Discussion

This and our previous study [21] indicate that the HIV-1 subtype G likely originated in Central Africa around the middle-late 1960s and began to be disseminated to Western and West-Central Africa from the middle 1970s onwards. Some of the subtype G strains disseminated out of Central Africa fueled secondary outbreaks that led to the origin of regional-specific subtype G clades. The major subtype G clades detected in our previous study in West Africa were the G_WA-I_ (that most probably emerged in Nigeria around the middle 1970s) and the G_WA-II_ (that most probably emerged in Togo or Ghana around the late 1970s) [21]. In the present study we identified a novel major clade (G_CV-PT_) that probably emerged between the late 1970s and the middle 1980s and circulates in Cape Verde and Portugal.

The G_CV-PT_ clade comprises 95% and 80% of HIV-1 subtype G *pol* sequences from Portugal and Cape Verde included in our study, respectively. Within the G_CV-PT_ radiation, most sequences from Portugal (73%) branched in a monophyletic subclade together with the CRF14_BG reference sequences, whereas the remaining Portuguese sequences branched at the base of the CRF14_BG-like subclade. This clearly indicates that the G_CV-PT_ clade is the parental subtype G lineage of the CRF14_BG variant and that the CRF14_BG clade is probably more prevalent in Portugal than the parental G_CV-PT_ clade, consistent with previous findings [25]. It is also important to note that a small fraction of G_CV-PT_ *pol* sequences from Cape Verde (15%) also branched within the CRF14_BG-like clade, indicating that this recombinant lineage not only circulates in Portugal and Spain, but also in Cape Verde. Full-length genome analyses of Cape Verdean HIV-1 subtype G *pol* sequences that branched within the CRF14_BG-like subclade should be performed to confirm this hypothesis.

The phylogeographic analyses that combined subtype G sequences of the G_CV-PT_ clade (with exception of the CRF14_BG-like lineage) and basal G_CA_ clades consistently pointed to Cape Verde as the most probable root location of the G_CV-PT_ clade (*PSP* = 0.68–0.76). When CRF14_BG-like sequences are included, the root location of the G_CV-PT_ clade was most probably placed in Portugal (*PSP* = 0.55–0.81). It has been shown that convenience sampling (particularly sampling heterogeneity) can obfuscate the accurate estimation of ancestral spatial locations based on standard phylogeographic continuous-time Markov chain implementation [49]. When CRF14_BG-like sequences are included, the number of Portuguese sequences (*n* = 104) far exceeds the number of Cape Verdean sequences (*n* = 48) within the G_CV-PT_ clade and such a larger sample from Portugal may results in the higher support for this location as the origin of that clade. Thus, according to the more balanced data sets the founder G_CV-PT_ ancestor probably moved from Central Africa to Cape Verde and later passed from Cape Verde to Portugal.

Whereas the inclusion of the CRF14_BG-like sequences has a great impact on estimation of the G_CV-PT_ ancestral root location, ancestral root ages were mainly influenced by the inclusion of basal G_CA_ clades. The median *T*_MRCA_ of the G_CV-PT_ clade was traced to the late 1970s when basal G_CA_ clades were included, and to the middle 1980s when those basal sequences were not included (S5 Table). Similarly, the *T*_MRCA_ of the CRF14_BG clade moved from the middle 1980s to the early 1990s when G_CA_ clades were removed from the analysis (S5 Table). This suggests that inclusion of basal lineages from Central Africa tend to produce slightly older internal node ages, although no significant changes are observed in the mean estimated substitution rates (S5 Table). This observation, however, should be interpreted with caution because those *T*_MRCA_ estimates displayed a considerable overlap of the confidence interval and thus should not be regarded as statistically different.

Regardless the precise root age, our phylogeographic analyses support a nearly simultaneous introduction and concurrent dissemination of the G_CV-PT_ clade in Cape Verde and Portugal. Our phylogeographic analyses based on balanced datasets suggest that the G_CV-PT_ clade started to be disseminated in Portugal only a couple of years later than the estimated introduction of the virus into Cape Verde. Of note, the estimated time-frame (1977–1984) for introduction and dissemination of the G_CV-PT_ clade in Cape Verde and Portugal was preceded by a phase of negative migratory outflow in Angola [50], associated to the exodus of thousands of Portuguese citizens of European and African ethnicity from Angola after the country independence in 1974. This may have fueled the chance exportation of the G_CV-PT_ ancestor strain from Angola into Cape Verde and its rapid dissemination to Portugal, thus suggesting that the global route of spread of the G_CV-PT_ clade was probably laid out along the colonial history ties, as has been previously demonstrated for the HIV-2 group A [49].

Despite the continuous and extensive migration of people between Angola, Cape Verde, and Portugal [28,51], subtype G strains sampled in those Portuguese-speaking countries retain a high phylogeographic structure with relative few viral exchanges among them. We have detected a total of: 1) four independent introductions of G_CA_ strains from Central Africa into Portugal, 2) three introductions of G_CA_ strains from Central Africa into Cape Verde, 3) three introductions of G_CV-PT_ strains from Cape Verde into Portugal, and 4) one G_CV-PT_ migration and five CRF14_BG introductions from Portugal into Cape Verde. Although the continuous viral exchanges among these countries may suppose a risk to the emergence of new country-specific subtype G lineages, most viral introductions seem to have failed to sustain new local subtype G epidemics with exception of the G_CV-PT_ founder strain.

According to our analysis, the G_CV-PT_ clade displayed a logistic population growth pattern characterized by an initial phase of exponential growth with a median rate of 0.52 year^-1^ (95% HPD: 0.32–0.77 year^-1^), followed by a decline in growth rate since the early 2000s. The median estimated logistic growth rate of the G_CV-PT_ clade was similar to that estimated for basal G_CA_ clades in Central Africa (0.47 year^-1^) [21] and the G_CU_ clade circulating in Cuba (0.55 year^-1^) [52]; but lower than those previously estimated for the G_WA-I_ (0. 75 year^-1^) and G_WA-II_ (0.95 year^-1^) clades circulating in continental West African countries [21] (S6 Table). The differential growth rates detected among different subtype G clades could be associated to clade-specific or ecological-specific differences in viral transmissibility. Further studies should be performed to understand whether the G_WA-I_ and G_WA-II_ clades introduced into continental West Africa displayed a higher intrinsic transmissibility or encountered more favorable epidemiological conditions for local and regional expansion than those disseminated within Central Africa, Cape Verde, Cuba and Portugal.

A previous study concluded that the CRF14_BG emerged in Portugal in the early 1990s and then spread to the North of Spain in late 1990s following the mobility of HIV-infected IDUs [27]. Our phylogeographic analyses indicate that the CRF14_BG clade probably arose in Portugal between the middle 1980s and the early 1990s, which is fully consistent with the previous estimation and with epidemiological data showing that CRF14_BG was already circulating in Lisbon in 1993 [27]. According to this estimate, the recombinant ancestor of the CRF14_BG clade was generated about five years after the estimated arrival of the parental G_CV-PT_ clade into Portugal, thus indicating a very rapid generation of BG recombinants in this country. After a period of local dissemination within Portugal, the CRF14_BG clade was dispersed not only from Portugal to Spain, but also probably to Cape Verde at multiple times.

In summary, this study reveals that most HIV-1 subtype G infections in Cape Verde and Portugal have resulted from the local dissemination of a single clade (here called G_CV-PT_) that probably emerged after a single migration of the virus out of Central Africa into Cape Verde between the late 1970s and the middle 1980s. Dispersion of the G_CV-PT_ clade seems to have been shaped by the historical and ongoing human population movements between Angola, Cape Verde and Portugal,. Our data also highlight that once introduced in Portugal, the G_CV-PT_ was disseminated in the local population and probably recombined with local preexisting subtype B variants, originating the CRF14_BG clade. These findings offer important insights to understanding the origin and current characteristics of the HIV-1 subtype G and CFR14_BG epidemics in Cape Verde and Portugal.

## Supporting Information

S1 Fig Analyses of phylogenetic signal and substitution saturation. (A—E) Likelihood maps of 10,000 random quartets made from every HIV-1 subtype G dataset used in this study as indicated in the figure. The triangles display the distribution (left) and percentage (right) of dots representing the likelihoods of the three possible tree topologies for a group of four sequences (quartets) randomly selected from the dataset. The tree-like, star-like and network-like phylogenetic signals are represented by the dots localized on the vertices, center and on the laterals, respectively. Fully resolved (tree-like) tree topologies ranged from 0.77 (CRF14-like-CV-PT) to 0.93 (G-CA + G-WA + G-CV-PT), thus indicating enough phylogenetic signal for consistent phylogenetic inferences in all datasets. (F—J) Substitution saturation plots of the datasets used in this study as depicted in the figure. The ordinate corresponds to the observed proportion of transitions (*s*, green) and transversions (*v*, blue) while the abscissa refers to the distance calculated using the GTR substitution model. The central lines of each plot correspond to the quadratic nonlinear regressions of the data. CA—Central Africa, WA—West Africa, CV—Cape Verde, PT—Portugal. All analyses indicated an absence of substitution saturation in the data set explored since the plots did not reach an evident plateau nor the transversions outnumbered transitions.
(PDF)

S1 Table GenBank accession numbers of HIV-1 subtype G *pol* sequences described in Table 1. (PDF)

S2 Table Distribution of HIV-1 subtype G *pol* sequences across major regional clades circulating in Central/West-Central Africa (G_CA_), West Africa (G_WA-I_ and G_WA-II_) and Cape Verde/Portugal (G_CV-PT_). AO/CD/CG: Angola/Democratic Republic of Congo/Republic of Congo. GA/GQ: Gabon/Equatorial Guinea. GH/TG: Ghana/Togo. PT/ES: Portugal/Spain.
(PDF)

S3 Table HIV-1 subtype G *pol* dataset used for Bayesian phylogeographic analysis. ^a^The number of subtype G *pol* fragments recovered from full-length HIV-1 CRF14_BG reference sequences is indicated in parenthesis. DRC: Democratic Republic of Congo.
(PDF)

S4 Table Best fit demographic model for HIV-1 G_CV-PT_ clade. Log marginal likelihood (ML) estimates for the logistic (Log), exponential (Expo) and expansion (Expa) growth demographic models obtained using the path sampling (PS) and stepping-stone sampling (SS) methods. The Log Bayes factor (BF) is the difference of the Log ML between of alternative (H1) and null (H0) models (H1/H0). Log BFs > 3 indicates that model H1 is more strongly supported by the data than model H0.
(PDF)

S5 Table Bayesian estimates of the age and root location of the most recent common ancestor (MRCA) of major HIV-1 subtype G (G_CV-PT_) and BG (CRF14_BG) clades circulating in Cape Verde and Portugal. ^a^ substitutions/site/year. CV: Cape Verde. PT: Portugal. TMRCA: time of the most recent common ancestor. PSP: posterior state probability.
(PDF)

S6 Table Evolutionary and demographic parameters estimated for major HIV-1 subtype G clades circulating in Central/West-Central Africa (G_CA_), West Africa (G_WA-I_ and G_WA-II_), Cuba (G_CU_) and Cape Verde/Portugal (G_CV-PT_). ^a^ Data from Delatorre et al [21]. ^b^ Data from Delatorre et al [52]. ^c^ Estimated at this study.
(PDF)
