# Supplementary figures and images for: Origin and Population Dynamics of a Novel HIV-1 Subtype G Clade Circulating in Cape Verde and Portugal
Source: PLoS One. 2015 May 20;10(5):e0127384. doi: 10.1371/journal.pone.0127384 (PMC4439163; doi:10.1371/journal.pone.0127384)

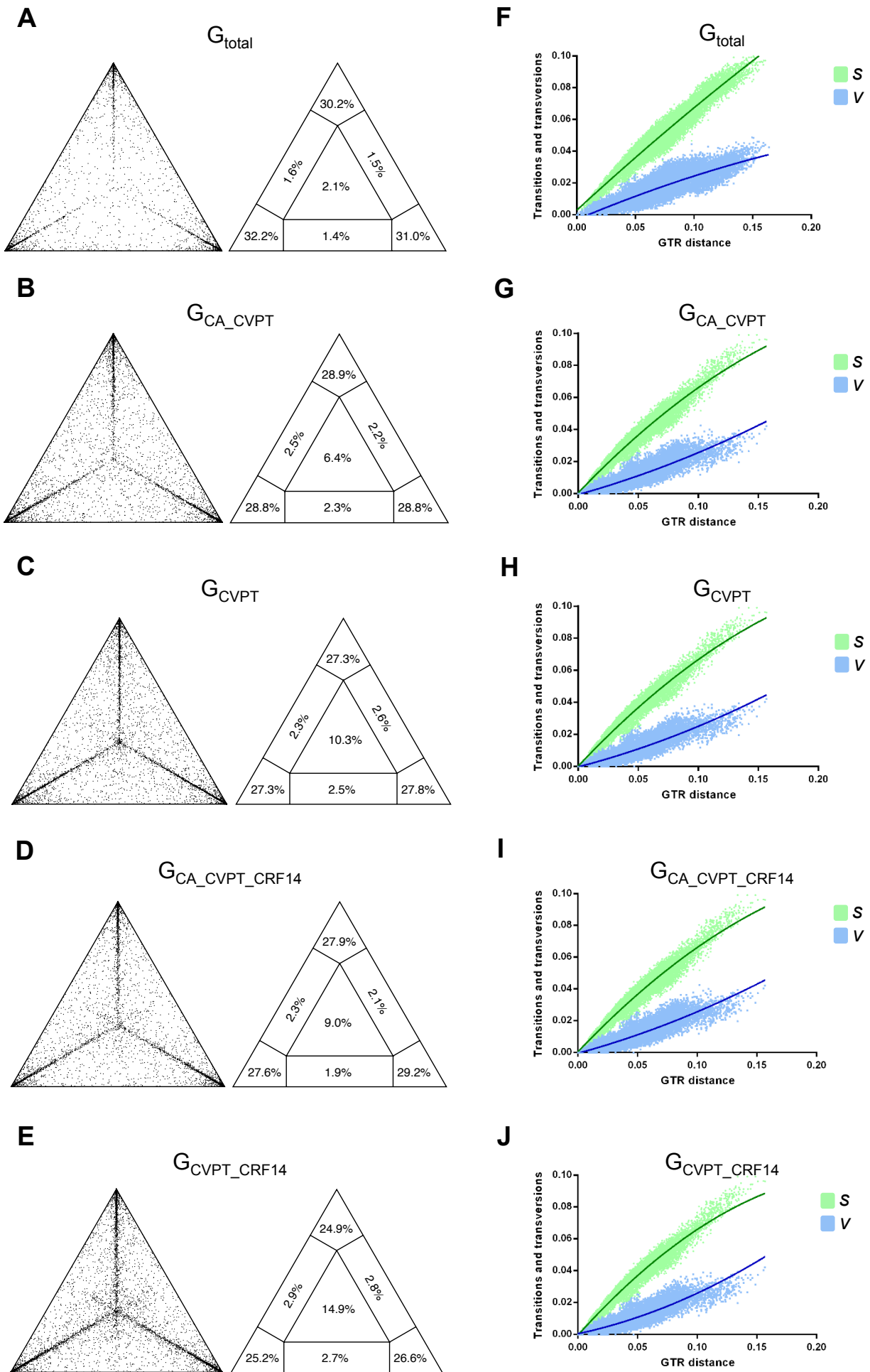

S1 Fig.

Supplement: S1 Fig — (A—E) Likelihood maps of 10,000 random quartets made from every HIV-1 subtype G dataset used in this study as indicated in the figure. The triangles display the distribution (left) and percentage (right) of dots representing the likelihoods of the three possible tree topologies for a group of four sequences (quartets) randomly selected from the dataset. The tree-like, star-like and network-like phylogenetic signals are represented by the dots localized on the vertices, center and on the laterals, respectively. Fully resolved (tree-like) tree topologies ranged from 0.77 (CRF14-like-CV-PT) to 0.93 (G-CA + G-WA + G-CV-PT), thus indicating enough phylogenetic signal for consistent phylogenetic inferences in all datasets. (F—J) Substitution saturation plots of the datasets used in this study as depicted in the figure. The ordinate corresponds to the observed proportion of transitions (s, green) and transversions (v, blue) while the abscissa refers to the distance calculated using the GTR substitution model. The central lines of each plot correspond to the quadratic nonlinear regressions of the data. CA—Central Africa, WA—West Africa, CV—Cape Verde, PT—Portugal. All analyses indicated an absence of substitution saturation in the data set explored since the plots did not reach an evident plateau nor the transversions outnumbered transitions. (PDF) [file pone.0127384.s001.pdf]
