# Supplementary material for: Origin and Population Dynamics of a Novel HIV-1 Subtype G Clade Circulating in Cape Verde and Portugal
Source: PLoS One. 2015 May 20;10(5):e0127384. doi: 10.1371/journal.pone.0127384 (PMC4439163; doi:10.1371/journal.pone.0127384)
Supplement: S1 Table — (PDF) [file pone.0127384.s002.pdf]

Subtype	Country of origin	Year	GenBank Accession Number
G	AO	1997	AF196695/AF212297
G	AO	2001	EU068224/EU068365
G	AO	2001	EU068235/EU068375
G	AO	2001	EU068262/EU068410
G	AO	2001	EU068272/EU068420
G	AO	2001	EU068302/EU068448
G	AO	2009	JQ616875
G	AO	2009	JQ616879
G	AO	2009	JQ616909
G	AO	2009	JN937056
G	AO	2010	JN937072
G	AO	2010	JN937088
G	AO	2010	JN937096
G	AO	2009	KF853775/KF853635
G	AO	2009	KF853819/KF853678
G	AO	2009	KF853843/KF853702
G	AO	2009	KF853847/KF853706
G	AO	2009	KF853848/KF853707
G	AO	2009	KF853858/KF853717
G	AO	2009	KF853891/KF853750
G	CD	1993	AF061642
G	CD	1996	AF084936
G	CD	2002	AM041000
G	CD	2002	AM041012
G	CD	2002	AM041028
G	CD	2002	AM041043
G	CD	2002	AM041046
G	CD	2007	FR666616
G	CD	2007	FR666627
G	CD	2007	FR666630
G	CD	2007	FR666643
G	CD	2007	FR666644
G	CG	2003	FM164894
G	CG	2003	FM164902
G	CG	2003	FM164906
G	CG	2003	FM164916
G	CG	2003	FM164923
G	CG	2003	FM164925
G	CG	2003	FM164898
G	CG	2003	FM164932
G	CM	1997	AJ286963
G	CM	1997	AM279373
G	CM	1998	AJ286964
G	CM	1998	AJ286965

G	CM	1999	AM279377
G	CM	1999	AM279378
G	CM	1999	AM279386
G	CM	2001	AY371121
G	CM	2001	FJ389367
G	CM	2001	GU207087
G	CM	2001	GU207114
G	CM	2004	FJ389363
G	CM	2004	FJ389364
G	CM	2004	FJ389365
G	CM	2004	FJ389366
G	CM	2006	FJ688263
G	CM	2006	JN639446
G	CM	2007	GU191553
G	CM	2007	GU191597
G	CM	2007	GU191639
G	CM	2007	HQ864365
G	CM	2007	HQ864366
G	CM	2007	HQ864367
G	CM	2007	HQ864368
G	CM	2007	JN639457
G	CM	2007	JN639458
G	CM	2008	HQ864369
G	CM	2009	JQ796107
G	CM	2009	JQ796146
G	CM	2010	JX140676
G	CM	2011	JQ796143
G	CM	2009	KF192120
G	CM	2009	KF192119
G	CM	2009	KF192118
G	CM	2009	KF192117
G	CM	2009	KF192116
G	CM	2011	KC350376
G	CM	2009	KC350338
G	CM	2010	KC350329
G	CM	2009	KC350320
G	CM	2010	KC350193
G	CM	2012	KF735836
G	CM	2009	KF751451
G	CM	2009	KF751449
G	CM	2009	KF751447
G	CM	2010	KF751446
G	CM	2009	KF751445
G	CM	2009	KF751444
G	CM	2010	KF751443
G	CM	2010	KF751442

G	CM	2009	KF751441
G	CM	2009	KF751440
G	CM	2010	KF751438
G	CM	2009	KF751437
G	CM	2009	KF751436
G	CM	2009	KF751435
G	CM	2011	KF751434
G	CM	2010	KF751433
G	CM	2009	KF751432
G	CM	2010	KF751431
G	CM	2010	KF751430
G	CM	2010	KF751429
G	GA	2000	AJ313401
G	GA	2000	AJ313412
G	GA	2008	HQ541916
G	GA	2008	HQ541926
G	GA	2008	HQ541939
G	GA	2008	HQ541943
G	GQ	2008	FN557306
G	GQ	2005	EU255342
G	GQ	2007	EU545193
G	GQ	2009	JX428555
G	BJ	2004	HF586667
G	BJ	2004	HF586670
G	BJ	2008	HQ267539
G	BJ	2008	HQ267546
G	BJ	2008	HQ267569
G	BJ	2008	HQ267577
G	BJ	2008	HQ267581
G	BJ	2008	HQ267582
G	BJ	2008	HQ267607
G	BJ	2008	HQ267617
G	BJ	2008	HQ267630
G	BJ	2009	HQ267631
G	BJ	2009	HQ267634
G	BJ	2009	HQ267655
G	BJ	2009	HQ267657
G	GH	2002	EU178021
G	GH	2002	EU178026
G	GH	2002	EU178067
G	GH	2003	AB231893
G	GH	2003	EF174563
G	GH	2007	HQ170613
G	GH	2008	GU969547
G	GH	2009	HQ667698
G	GH	2010	KF907218

G	NG	1992	U88826
G	NG	1999	AY181068
G	NG	1999	AY181069
G	NG	1999	AY181070
G	NG	1999	AY181071
G	NG	1999	AY181072
G	NG	1999	AY181074
G	NG	1999	AY181075
G	NG	1999	AY181076
G	NG	1999	AY181078
G	NG	1999	AY181080
G	NG	1999	AY181081
G	NG	1999	AY181082
G	NG	2001	DQ013269
G	NG	2001	DQ013270
G	NG	2001	DQ013272
G	NG	2001	DQ013275
G	NG	2001	DQ013278
G	NG	2001	DQ013279
G	NG	2001	DQ013280
G	NG	2001	DQ168573
G	NG	2001	DQ168579
G	NG	2006	HQ843509
G	NG	2006	HQ843517
G	NG	2006	HQ843522
G	NG	2007	HQ843511
G	NG	2007	HQ843516
G	NG	2007	HQ843523
G	NG	2007	HQ843524
G	NG	2007	HQ843530
G	NG	2007	HQ843531
G	NG	2007	HQ843534
G	NG	2007	HQ843535
G	NG	2007	HQ843539
G	NG	2007	HQ843540
G	NG	2007	HQ843541
G	NG	2007	HQ843545
G	NG	2007	HQ843546
G	NG	2007	HQ843547
G	NG	2007	HQ843548
G	NG	2007	HQ843550
G	NG	2007	HQ843551
G	NG	2007	HQ843552
G	NG	2007	HQ843557
G	NG	2007	HQ843558
G	NG	2007	HQ843561

G	NG	2007	HQ843565
G	NG	2007	HQ843566
G	NG	2007	HQ843571
G	NG	2007	HQ843573
G	NG	2007	HQ843578
G	NG	2007	HQ843579
G	NG	2007	HQ843584
G	NG	2007	HQ843585
G	NG	2007	HQ843587
G	NG	2007	HQ843592
G	NG	2007	HQ843593
G	NG	2007	HQ843597
G	NG	2007	HQ843598
G	NG	2007	HQ843603
G	NG	2007	HQ843604
G	NG	2007	HQ843610
G	NG	2007	HQ843611
G	NG	2007	HQ843612
G	NG	2007	HQ843613
G	NG	2007	HQ843615
G	NG	2007	HQ843618
G	NG	2007	HQ843619
G	NG	2007	HQ843620
G	NG	2007	HQ843621
G	NG	2007	HQ843622
G	NG	2007	HQ843624
G	NG	2007	HQ843625
G	NG	2007	HQ843630
G	NG	2007	HQ843632
G	NG	2007	HQ843633
G	NG	2007	HQ843634
G	NG	2007	HQ843635
G	NG	2007	HQ843637
G	NG	2007	HQ843638
G	NG	2007	HQ843640
G	NG	2007	HQ843643
G	NG	2007	HQ843648
G	NG	2007	HQ843650
G	NG	2007	HQ843652
G	NG	2007	HQ843655
G	NG	2007	HQ843657
G	NG	2007	HQ843661
G	NG	2007	HQ843667
G	NG	2007	HQ843668
G	NG	2007	HQ843674
G	NG	2007	HQ843676

G	NG	2007	HQ843677
G	NG	2007	HQ843679
G	NG	2007	HQ843680
G	NG	2007	HQ993979
G	NG	2007	HQ993982
G	NG	2007	HQ993986
G	NG	2007	HQ993987
G	NG	2007	HQ993994
G	NG	2007	HQ993996
G	NG	2007	HQ994000
G	NG	2007	HQ994004
G	NG	2007	HQ994010
G	NG	2007	HQ994011
G	NG	2007	HQ994014
G	NG	2007	HQ994016
G	NG	2007	HQ994017
G	NG	2007	HQ994018
G	NG	2007	HQ994019
G	NG	2007	HQ994026
G	NG	2007	HQ994030
G	NG	2007	HQ994031
G	NG	2007	HQ994033
G	NG	2007	HQ994036
G	NG	2007	HQ994037
G	NG	2007	HQ994042
G	NG	2007	HQ994046
G	NG	2007	HQ994049
G	NG	2007	HQ994052
G	NG	2007	HQ994056
G	NG	2007	HQ994057
G	NG	2007	HQ994075
G	NG	2007	HQ994079
G	NG	2007	HQ994085
G	NG	2007	HQ994094
G	NG	2007	HQ994095
G	NG	2007	HQ994098
G	NG	2007	HQ994104
G	NG	2007	HQ994114
G	NG	2007	HQ994115
G	NG	2007	HQ994125
G	NG	2007	HQ994130
G	NG	2007	HQ994139
G	NG	2007	HQ994144
G	NG	2007	HQ994146
G	NG	2007	HQ994149
G	NG	2007	HQ994150

G	NG	2007	HQ994158
G	NG	2007	HQ994159
G	NG	2007	HQ994160
G	NG	2007	JQ480191
G	NG	2007	JQ480195
G	NG	2007	JQ480198
G	NG	2007	JQ480199
G	NG	2007	JQ480200
G	NG	2007	JQ480201
G	NG	2007	JQ480202
G	NG	2007	JQ480206
G	NG	2008	HQ845921
G	NG	2008	HQ845926
G	NG	2008	HQ845927
G	NG	2008	HQ845928
G	NG	2008	JN132350
G	NG	2008	JN132355
G	NG	2008	JN248582
G	NG	2009	HQ845930
G	NG	2009	HQ845934
G	NG	2009	HQ845935
G	NG	2009	HQ845936
G	NG	2009	HQ845937
G	NG	2009	JN132365
G	NG	2009	JN132367
G	NG	2009	JN132368
G	NG	2009	JN132370
G	NG	2009	JN132371
G	NG	2009	JN132372
G	NG	2009	JN132373
G	NG	2009	JN132378
G	NG	2009	JN248584
G	NG	2009	JN248586
G	NG	2009	JN248591
G	NG	2009	JN248593
G	NG	2010	HQ845939
G	NG	2010	HQ845940
G	NG	2010	HQ845941
G	NG	2010	HQ845944
G	NG	2010	HQ845945
G	NG	2010	HQ845947
G	NG	2010	HQ845948
G	NG	2010	HQ845950
G	NG	2010	HQ845954
G	NG	2010	HQ845957
G	NG	2010	KF241510

G	NG	2010	KF241509
G	NG	2010	KF241508
G	NG	2011	KF241505
G	NG	2010	KF241504
G	NG	2010	KF241502
G	NG	2010	KF241501
G	NG	2011	KF241500
G	NG	2007	KF241495
G	NG	2011	KF241488
G	NG	2011	KF241487
G	NG	2011	KF241485
G	NG	2011	KF241484
G	NG	2010	KF241481
G	NG	2008	KF241475
G	NG	2008	KF241474
G	NG	2008	KF241470
G	NG	2008	KF241468
G	NG	2008	KF241467
G	NG	2008	KF241463
G	NG	2009	KF241460
G	NG	2013	KC755269
G	NG	2013	KC755265
G	NG	2013	KC755263
G	NG	2013	KC755262
G	NG	2013	KC755261
G	NG	2013	KC755260
G	NG	2013	KC755258
G	NG	2013	KC755257
G	NG	2013	KC755255
G	NG	2013	KC755253
G	NG	2013	KC755252
G	NG	2013	KC755250
G	NG	2013	KC755249
G	NG	2013	KC755247
G	NG	2013	KC755244
G	NG	2013	KC755241
G	NG	2013	KC755240
G	NG	2013	KC755239
G	NG	2013	KC755238
G	SN	1998	AJ286982
G	SN	2001	FN599705
G	SN	2004	FM210696
G	SN	2004	FM210697
G	SN	2004	FM210704
G	SN	2004	FM210706
G	SN	2004	FM210708

G	SN	2005	FN599788
G	SN	2009	HM002537
G	SN	2009	JN673700
G	SN	2009	JN673705
G	SN	2010	JN673706
G	TG	2006	FM955693
G	TG	2006	FM955700
G	TG	2006	FM955702
G	TG	2006	FM955711
G	TG	2006	FM955712
G	TG	2006	FM955715
G	TG	2006	FM955716
G	TG	2006	FM955721
G	TG	2006	FM955734
G	TG	2006	FM955738
G	TG	2008	FR715270
G	TG	2008	FR715281
G	TG	2008	FR715290
G	TG	2011	KC350318
G	TG	2009	KC350310
G	TG	2011	KC350301
G	TG	2009	KC350216
G	TG	2009	KC350214
G	TG	2009	KC350208
G	TG	2010	KC350205
G	TG	2010	KC350181
G	TG	2010	KC350115
G	TG	2009	KC350114
G	TG	2010	KC350113
G	TG	2009	KC350112
G	TG	2010	KC350111
G	TG	2010	KC350110
G	TG	2009	KC350109
G	CV	2010	KJ395597
G	CV	2010	KJ395602
G	CV	2010	KJ395603
G	CV	2010	KJ395604
G	CV	2010	KJ395605
G	CV	2010	KJ395606
G	CV	2010	KJ395607
G	CV	2010	KJ395608
G	CV	2010	KJ395609
G	CV	2010	KJ395610
G	CV	2010	KJ395615
G	CV	2010	KJ395621
G	CV	2010	KJ395623

G	CV	2010	KJ395630
G	CV	2010	KJ395635
G	CV	2010	KJ395639
G	CV	2010	KJ395640
G	CV	2010	KJ395642
G	CV	2010	KJ395646
G	CV	2010	KJ395649
G	CV	2010	KJ395658
G	CV	2011	KJ395665
G	CV	2011	KJ395668
G	CV	2011	KJ395669
G	CV	2011	KJ395670
G	CV	2011	KJ395671
G	CV	2011	KJ395672
G	CV	2011	KJ395673
G	CV	2011	KJ395675
G	CV	2011	KJ395677
G	CV	2011	KJ395680
G	CV	2011	KJ395681
G	CV	2011	KJ395684
G	CV	2011	KJ395688
G	CV	2011	KJ395689
G	CV	2011	KJ395690
G	CV	2011	KJ395691
G	CV	2011	KJ395692
G	CV	2011	KJ395702
G	CV	2011	KJ395703
G	CV	2011	KJ395704
G	CV	2011	KJ395708
G	CV	2011	KJ395709
G	CV	2011	KJ395710
G	CV	2011	KJ395711
G	CV	2011	KJ395715
G	CV	2010	KJ395619
G	CV	2010	KJ395632
G	CV	2005	JF267440/JF267466
G	CV	2005	JF267442/JF267468
G	CV	2005	JF267443/JF267469
G	CV	2005	JF267445/JF267472
G	CV	2005	JF267446/JF267473
G	CV	2005	JF267448/JF267475
G	CV	2005	JF267450/JF267477
G	CV	2005	JF267459/JF267485
G	CV	2005	JF267461/JF267486
G	CV	2005	JF267457/JF267489
G	CV	2005	JF267458/JF267490

G	PT	2003	DQ877867
G	PT	2003	GQ398828
G	PT	2004	GQ398850
G	PT	2004	GQ398851
G	PT	2004	GQ398857
G	PT	2005	GQ398866
G	PT	2005	GQ398880
G	PT	2004	GQ398892
G	PT	2003	GQ398906
G	PT	2004	GQ398969
G	PT	2003	GQ398983
G	PT	2003	GQ398994
G	PT	2004	GQ399006
G	PT	2004	GQ399022
G	PT	2005	GQ399108
G	PT	2004	GQ399178
G	PT	2004	GQ399192
G	PT	2003	GQ399196
G	PT	2004	GQ399239
G	PT	2003	GQ399279
G	PT	2004	GQ399283
G	PT	2003	GQ399331
G	PT	2004	GQ399349
G	PT	2005	GQ399357
G	PT	2003	GQ399358
G	PT	2005	GQ399365
G	PT	2003	GQ399375
G	PT	2003	GQ399441
G	PT	2005	GQ399521
G	PT	2003	GQ399618
G	PT	2003	GQ399671
G	PT	2004	GQ399678
G	PT	2003	GQ399783
G	PT	2005	GQ399797
G	PT	2004	GQ399808
G	PT	2003	GQ399830
G	PT	2003	GQ399843
G	PT	2003	GQ399908
G	PT	2003	GQ399921
G	PT	2005	GQ399924
G	PT	2005	GQ399947
G	PT	2003	GQ399962
G	PT	2003	GQ400013
G	PT	2003	GQ400076
G	PT	2004	GQ400083
G	PT	2004	GQ400111

G	PT	2005	GQ400115
G	PT	2004	GQ400126
G	PT	2005	GQ400130
G	PT	2004	GQ400133
G	PT	2004	GQ400171
G	PT	2003	GQ400191
G	PT	2005	GQ400213
G	PT	2004	GQ400215
G	PT	2004	GQ400218
G	PT	2003	GQ400223
G	PT	2003	GQ400252
G	PT	2004	GQ400287
G	PT	2004	GQ400290
G	PT	2004	GQ400323
G	PT	2003	GQ400356
G	PT	2003	GQ400357
G	PT	2005	GQ400368
G	PT	2003	GQ400386
G	PT	2004	GQ400393
G	PT	2004	GQ400421
G	PT	2003	GQ400448
G	PT	2004	GQ400469
G	PT	2003	GQ400471
G	PT	2003	HM135470
G	PT	2003	HM135471
G	PT	2003	HM135472
G	PT	2003	HM135473
G	PT	2003	HM135474
G	PT	2003	HM135475
G	PT	2003	HM135476
G	PT	2003	HM135477
G	PT	2003	HM135478
G	PT	2003	HM135479
G	PT	2003	HM135480
G	PT	2003	HM135481
G	PT	2003	HM135482
G	PT	2003	HM135483
G	PT	2003	HM135485
G	PT	2003	HM135487
G	PT	2003	HM135488
G	PT	2004	HM135489
G	PT	2004	HM135492
G	PT	2004	HM135493
G	PT	2004	HM135494
G	PT	2004	HM135495
G	PT	2004	HM135496

G	PT	2004	HM135497
G	PT	2004	HM135499
G	PT	2004	HM135500
G	PT	2004	HM135501
G	PT	2004	HM135502
G	PT	2004	HM135505
G	PT	2004	HM135506
G	PT	2004	HM135507
G	PT	2004	HM135509
G	PT	2008	HM135510
G	PT	2008	HM135511
G	PT	2008	HM135512
G	PT	1998	AY612637
G	PT	1998	FR846409
G	PT	1999	FR846410
14_BG	ES	1999	AF423756
14_BG	ES	1999	AF423757
14_BG	ES	2000	AF423758
14_BG	ES	2000	AF423759
14_BG	ES	2000	AF450096
14_BG	ES	2000	AF450097
14_BG	ES	2004	FJ670517
14_BG	ES	2004	FJ670518
14_BG	ES	2005	FJ670522
14_BG	ES	2005	FJ670528
14_BG	PT	2000	GU230137
14_BG	PT	2000	GU230138
