# Supplementary material for: Origin and Population Dynamics of a Novel HIV-1 Subtype G Clade Circulating in Cape Verde and Portugal
Source: PLoS One. 2015 May 20;10(5):e0127384. doi: 10.1371/journal.pone.0127384 (PMC4439163; doi:10.1371/journal.pone.0127384)
Supplement: S2 Table — AO/CD/CG: Angola/Democratic Republic of Congo/Republic of Congo. GA/GQ: Gabon/Equatorial Guinea. GH/TG: Ghana/Togo. PT/ES: Portugal/Spain. (PDF) [file pone.0127384.s003.pdf]

S2 Table. Distribution of HIV-1 subtype G *pol* sequences across major regional clades circulating in Central/West-Central Africa (G_{CA}), West Africa (G_{WA-I} and G_{WA-II}) and Cape Verde/Portugal (G_{CV-PT}).

Country	*N*	G_{CA}	G_{WA-I}	G_{WA-II}	G_{CV-PT}
AO/CD/CG	40	36 (90%)	-	-	4 (10%)
Cameroon	62	32 (52%)	23 (37%)	7 (11%)	-
GA/GQ	10	5 (50%)	2 (20%)	3 (30%)	-
Benin	15	3 (20%)	7 (47%)	5 (33%)	-
Cape Verde	60	2 (3%)	10 (17%)	-	48 (80%)
GH/TG	37	2 (5%)	4 (11%)	31 (84%)	-
Nigeria	223	-	175 (78%)	48 (22%)	-
Senegal	12	5 (42%)	6 (50%)	1 (8%)	-
Portugal (G)	107	5 (5%)	-	-	102 (95%)
PT/ES (CRF14)	12	-	-	-	12 (100%)

AO/CD/CG: Angola/Democratic Republic of Congo/Republic of Congo. GA/GQ: Gabon/Equatorial Guinea. GH/TG: Ghana/Togo. PT/ES: Portugal/Spain.
