# Supplementary material for: Origin and Population Dynamics of a Novel HIV-1 Subtype G Clade Circulating in Cape Verde and Portugal
Source: PLoS One. 2015 May 20;10(5):e0127384. doi: 10.1371/journal.pone.0127384 (PMC4439163; doi:10.1371/journal.pone.0127384)
Supplement: S3 Table — aThe number of subtype G pol fragments recovered from full-length HIV-1 CRF14_BG reference sequences is indicated in parenthesis. DRC: Democratic Republic of Congo. (PDF) [file pone.0127384.s004.pdf]

S3 Table. HIV-1 subtype G *pol* dataset used for Bayesian phylogeographic analyses.

Cade/subclade	Region	Country	Location	*N*	Sampling interval
G_{CA}	West Central Africa	Cameroon	CM	32	1997-2012
		Gabon	GA/GQ	4	2000-2008
		Equatorial Guinea		1	2009
	Central Africa	Angola	AO/CD/CG	20	1997-2010
		DRC		12	1993-2007
		Republic of Congo		8	2003
G_{CVPT}	West Africa	Cape Verde	CV	41	2005-2011
	Europe	Portugal	PT	24	1998-2008
CFR14_BG-like	West Africa	Cape Verde	CV	7	2005-2011
	Europe	Portugal	PT	80 (2)[a]	2000-2008
		Spain	ES	10 (10)[a]	1999-2005

[a] The number of subtype G *pol* fragments recovered from full-length HIV-1 CRF14_BG reference sequences is indicated in parenthesis. DRC: Democratic Republic of Congo.
