# Supplementary material for: Origin and Population Dynamics of a Novel HIV-1 Subtype G Clade Circulating in Cape Verde and Portugal
Source: PLoS One. 2015 May 20;10(5):e0127384. doi: 10.1371/journal.pone.0127384 (PMC4439163; doi:10.1371/journal.pone.0127384)
Supplement: S4 Table — Log marginal likelihood (ML) estimates for the logistic (Log), exponential (Expo) and expansion (Expa) growth demographic models obtained using the path sampling (PS) and stepping-stone sampling (SS) methods. The Log Bayes factor (BF) is the difference of the Log ML between of alternative (H1) and null (H0) models (H1/H0). Log BFs > 3 indicates that model H1 is more strongly supported by the data than model H0. (PDF) [file pone.0127384.s005.pdf]

S4 Table. Best fit demographic model for HIV-1 G_{CV-PT} clade.

Model	PS Log ML	Models compared	Log BF	SS Log ML	Models compared	Log BF
Log	**-7199.9**	-	-	**-7200.2**	-	-
Expo	-7211.4	Log/Expo	11.5	-7211.6	Log/Expo	11.4
Expa	-7222.6	Log/Expa	22.7	-7222.9	Log/Expa	22.7

Log marginal likelihood (ML) estimates for the logistic (Log), exponential (Expo) and expansion (Expa) growth demographic models obtained using the path sampling (PS) and stepping-stone sampling (SS) methods. The Log Bayes factor (BF) is the difference of the Log ML between of alternative (H1) and null (H0) models (H1/H0). Log BFs > 3 indicates that model H1 is more strongly supported by the data than model H0.
