# Supplementary material for: Origin and Population Dynamics of a Novel HIV-1 Subtype G Clade Circulating in Cape Verde and Portugal
Source: PLoS One. 2015 May 20;10(5):e0127384. doi: 10.1371/journal.pone.0127384 (PMC4439163; doi:10.1371/journal.pone.0127384)
Supplement: S5 Table — a substitutions/site/year. CV: Cape Verde. PT: Portugal. TMRCA: time of the most recent common ancestor. PSP: posterior state probability. (PDF) [file pone.0127384.s006.pdf]

S5 Table. Bayesian estimates of the age and root location of the most recent common ancestor (MRCA) of major HIV-1 subtype G (G_{CV-PT}) and BG (CRF14_BG) clades circulating in Cape Verde and Portugal.

Dataset	Sampling interval	Substitution rate[a]	G_{CVPT} Root location	G_{CVPT} T_{MRCA}	CRF14_BG Root location	CRF14_BG T_{MRCA}
G_{CA_CVPT}	1993-2012	1.6×10^{-3} (1.5-1.9×10^{-3})	CV (*PSP* = 0.68)	1977 (1972-1982)	-	-
G_{CVPT}	1998-2011	1.6×10^{-3} (1.5-1.9×10^{-3})	CV (*PSP* = 0.76)	1984 (1979-1989)	-	-
$G_{CA\_CVPT\_CRF14}$	1993-2012	1.8×10^{-3} (1.5-2.2×10^{-3})	PT (*PSP* = 0.55)	1979 (1973-1984)	PT (*PSP* = 1)	1986 (1982-1991)
G_{CVPT_CRF14}	1998-2011	1.8×10^{-3} (1.5-2.3×10^{-3})	PT (*PSP* = 0.81)	1984 (1976-1991)	PT (*PSP* = 1)	1991 (1988-1994)

[a] substitutions/site/year. CV: Cape Verde. PT: Portugal. T_{MRCA}: time of the most recent common ancestor. *PSP*: posterior state probability.
