# Supplementary material for: Origin and Population Dynamics of a Novel HIV-1 Subtype G Clade Circulating in Cape Verde and Portugal
Source: PLoS One. 2015 May 20;10(5):e0127384. doi: 10.1371/journal.pone.0127384 (PMC4439163; doi:10.1371/journal.pone.0127384)
Supplement: S6 Table — a Data from Delatorre et al [21]. b Data from Delatorre et al [52]. c Estimated at this study. (PDF) [file pone.0127384.s007.pdf]

S6 Table. Evolutionary and demographic parameters estimated for major HIV-1 subtype G clades circulating in Central/West-Central Africa (G_{CA}), West Africa (G_{WA-I} and G_{WA-II}), Cuba (G_{CU}) and Cape Verde/Portugal (G_{CV-PT}).

Clade	*N*	Sampling interval	T_{MRCA}	Logistic growth rate
G_{CA}[a]	60	1993-2011	1975 (1970-1981)	0.47 (0.33-0.63)
G_{WA-I}[a]	167	1999-2010	1978 (1970-1983)	0.75 (0.56-0.98)
G_{WA-II}[a]	72	1992-2010	1984 (1980-1987)	0.95 (0.68-1.30)
G_{CU}[b]	26	1999-2011	1988 (1976-1995)	0.55 (0.24-0.91)
G_{CV-PT}[c]	67	1998-2011	1984 (1980-1987)	0.52 (0.32-0.77)

[a] Data from Delatorre *et al* [21]. [b] Data from Delatorre *et al* [52]. [c] Estimated at this study.
